# Pirfenidone in Heart Failure with Preserved Ejection Fraction—Rationale and Design of the PIROUETTE Trial

**DOI:** 10.1007/s10557-019-06876-y

**Published:** 2019-05-08

**Authors:** Gavin A. Lewis, Erik B. Schelbert, Josephine H. Naish, Emma Bedson, Susanna Dodd, Helen Eccleson, Dannii Clayton, Beatriz Duran Jimenez, Theresa McDonagh, Simon G. Williams, Anne Cooper, Colin Cunnington, Fozia Zahir Ahmed, Rajavarma Viswesvaraiah, Stuart Russell, Stefan Neubauer, Paula R. Williamson, Christopher A. Miller

**Affiliations:** 1 Division of Cardiovascular Sciences, School of Medical Sciences, Faculty of Biology, Medicine and Health, Manchester Academic Health Science Centre, University of Manchester, Oxford Road, Manchester, M13 9PL UK; 2 Manchester University NHS Foundation Trust, Southmoor Road, Wythenshawe, Manchester, M23 9LT UK; 3 Department of Medicine, University of Pittsburgh School of Medicine, Pittsburgh, PA USA; 4 UPMC Cardiovascular Magnetic Resonance Center, Heart and Vascular Institute, Pittsburgh, PA USA; 5 Clinical and Translational Science Institute, University of Pittsburgh, Pittsburgh, PA USA; 6 Clinical Trials Research Centre, 2nd Floor – Institute in the Park, Alder Hey Children’s NHS Foundation Trust, University of Liverpool, Member of Liverpool Health Partners, Liverpool, L12 2AP UK; 7 Department of Biostatistics, University of Liverpool, Member of Liverpool Health Partners, Block F, Waterhouse Bld, 1-5 Brownlow Street, Liverpool, L69 3GL UK; 8 King’s College Hospital, Denmark Hill, London, SE5 9RS UK; 9 Salford Royal NHS Foundation Trust, Stott Lane, Salford, M6 8HD UK; 10 Stockport NHS Foundation Trust, Stepping Hill Hospital, Poplar Grove, Hazel Grove, Stockport, SK2 7JE UK; 11 East Cheshire NHS Trust, Victoria Road, Macclesfield, SK10 3BL UK; 12 Division of Cardiovascular Medicine, Radcliffe Department of Medicine, University of Oxford, Oxford, UK; 13 Wellcome Centre for Cell-Matrix Research, Division of Cell-Matrix Biology and Regenerative Medicine, School of Biology, Faculty of Biology, Medicine and Health, Manchester Academic Health Science Centre, University of Manchester, Oxford Road, Manchester, M13 9PT UK

**Keywords:** Fibrosis, Heart failure, Magnetic resonance imaging (MRI)

## Abstract

**Background:**

The PIROUETTE (PIRfenidOne in patients with heart failUre and preserved lEfT venTricular Ejection fraction) trial is designed to evaluate the efficacy and safety of the anti-fibrotic pirfenidone in patients with chronic heart failure and preserved ejection fraction (HFpEF) and myocardial fibrosis. HFpEF is a diverse syndrome associated with substantial morbidity and mortality. Myocardial fibrosis is a key pathophysiological mechanism of HFpEF and myocardial fibrotic burden is strongly and independently associated with adverse outcome. Pirfenidone is an oral anti-fibrotic agent, without haemodynamic effect, that leads to regression of myocardial fibrosis in preclinical models. It has proven clinical effectiveness in pulmonary fibrosis.

**Methods:**

The PIROUETTE trial is a randomised, double-blind, placebo-controlled phase II trial evaluating the efficacy and safety of 52 weeks of treatment with pirfenidone in patients with chronic HFpEF (symptoms and signs of heart failure, left ventricular ejection fraction ≥ 45%, elevated natriuretic peptides [BNP ≥ 100 pg/ml or NT-proBNP ≥ 300 pg/ml; or BNP ≥ 300 pg/ml or NT-proBNP ≥ 900 pg/ml if in atrial fibrillation]) and myocardial fibrosis (extracellular matrix (ECM) volume ≥ 27% measured using cardiovascular magnetic resonance). The primary outcome measure is change in myocardial ECM volume. A sub-study will investigate the relationship between myocardial fibrosis and myocardial energetics, and the impact of pirfenidone, using ^31^phosphorus magnetic resonance spectroscopy.

**Discussion:**

PIROUETTE will determine whether pirfenidone is superior to placebo in relation to regression of myocardial fibrosis and improvement in myocardial energetics in patients with HFpEF and myocardial fibrosis (NCT02932566).

**Clinical Trial Registration:**

clinicaltrials.gov (NCT02932566) https://clinicaltrials.gov/ct2/show/NCT02932566

**Electronic supplementary material:**

The online version of this article (10.1007/s10557-019-06876-y) contains supplementary material, which is available to authorized users.

## Introduction

As highlighted in recent commentaries, there is a disconnect between phase II and phase III drug trials in heart failure (HF); despite often promising phase II results, most phase III trials prove neutral or negative [1–3]. The reasons for this include a lack of understanding and identification of prognostically important pathophysiological mechanisms, failure of therapies to target these underlying mechanisms, non-specific phase II end points that are not reflective of disease pathway modulation and a one-size-fits-all approach that does not take account of pathophysiological heterogeneity. It is with these factors in mind that the PIROUETTE trial has been designed.

### Heart Failure with Preserved Ejection Fraction

Heart failure (HF) affects approximately 1–2% of the adult population in developed countries [4, 5]. Potentially up to one-half of HF patients have a preserved left ventricular (LV) ejection fraction (HFpEF), and the prevalence of HFpEF is rising as the population ages [6]. Despite the high associated morbidity and mortality, there remains no therapy with regulatory approval to reduce morbidity and mortality [7–14].

### Myocardial Fibrosis

Extracellular matrix (ECM) expansion secondary to excess collagen accumulation (i.e. myocardial fibrosis) is consistently demonstrated on a group level in myocardial tissue from patients with HFpEF, and there are considerable data demonstrating both the potential for myocardial ECM to have a primary aetiological role in HFpEF, and the adverse impact that ECM expansion has on myocardial mechanical, electrical and microvascular function [15–21]. Notably, however, histological and imaging studies have shown that myocardial fibrosis is not universal in HFpEF, with approximately one-third to one-half of patients having normal measures of myocardial fibrosis [15, 22].

Importantly, following previous smaller studies, Schelbert et al. showed myocardial ECM volume, measured using cardiovascular magnetic resonance (CMR) imaging (see below), was strongly associated with adverse outcome on multivariable analysis in a large cohort of patients (*n* = 410) with HFpEF or at risk for HFpEF (B-type natriuretic peptide (BNP) > 100 pg/ml but no clinical HF), with a clear “dose-response” relationship between ECM volume and outcome [22, 23].

Furthermore, there is human histological evidence that myocardial fibrosis is reversible, and, fibrosis regression appears to be most prominent in patients with a greater burden of myocardial fibrosis at baseline [24–27].

### Measurement of Myocardial Fibrosis

CMR imaging provides straightforward, robust, well-validated, accurate and highly reproducible quantification of myocardial ECM volume, and can detect clinical reversal of myocardial fibrosis [28–32]. In contrast, circulating collagen markers are not specific to the heart, being confounded by numerous factors such as renal function [33]. Similarly, echocardiographic variables are not specific for myocardial biological processes [19, 34].

### Rationale for Pirfenidone

Pirfenidone is an orally bioavailable, small molecule anti-fibrotic agent, with proven clinical effectiveness in idiopathic pulmonary fibrosis for which it is licenced in Europe and the USA [35–37].

The action of pirfenidone has been investigated across a range of preclinical models of fibrosis in the lung, liver, kidney and heart (including models of hypertension, diabetes, pressure overload and infarction), and human in vitro work [38–40]. In keeping with the findings in other organs, pirfenidone, in a dose- and time-dependent manner, inhibits cardiac fibroblast synthesis and secretion of TGF-β1 [41–48]. Via this mechanism, and also directly, pirfenidone inhibits the proliferation of cardiac fibroblasts, reduces their migratory ability and inhibits myofibroblast differentiation [41–48]. Furthermore, it has been shown to normalise ratios of myocardial matrix metalloproteinases (MMPs) and tissue inhibitors of metalloproteinases (TIMPs), improve myocardial renin-angiotensin system imbalance via activation of liver X receptor-α expression and enhance cardiac fibroblast synthesis and secretion of IL-10, an anti-fibrotic cytokine [41, 49]. As a result of these anti-fibrotic effects, pirfenidone is associated with an absolute decrease in LV collagen volume fraction of up to 6.5% in preclinical models, which is associated with improved LV function (systolic and diastolic variables) and decreased susceptibility to arrhythmias [42, 45–50]. Importantly from a mechanistic perspective for the PIROUETTE trial, pirfenidone does not have a haemodynamic effect.

Pirfenidone has proven to be safe and well tolerated in patients with pulmonary fibrosis in randomised controlled trials (RCT) and post-marketing surveillance [35–37, 51]. Gastrointestinal (nausea, dyspepsia, anorexia) and skin (rash)-related adverse events are more common with pirfenidone than with placebo, but they are generally mild and without clinically significant consequences. In the largest and most recent RCT, gastrointestinal and skin side effects led to treatment discontinuation in 2.2% and 2.9%, respectively, compared to 1.1% and 0.4% with placebo [36]. Clinically significant elevations in liver aminotransferase levels occurred more frequently with pirfenidone than with placebo (2.9% versus 0.7% respectively) but they were reversible and did not have clinically significant consequences.

## Trial Design and Methods

PIROUETTE is a randomised, double-blind, placebo-controlled phase II trial designed to evaluate the efficacy and safety of pirfenidone in patients with HFpEF and myocardial fibrosis. The hypothesis is that pirfenidone will target a fundamental, prognostically important underlying pathophysiological mechanism of HFpEF, i.e. myocardial fibrosis, in individual HFpEF patients with evidence of myocardial fibrosis, leading to regression of myocardial fibrosis. If true, it is hypothesised that this will lead onto improvements in cardiac structure and function, fluid status and quality of life, and thus, ultimately, translate into improved outcome. The trial was designed by the research team. The trial has been registered (NCT02932566).

### Study Objectives

The primary objective of this study is to evaluate whether pirfenidone compared to placebo leads to regression of myocardial fibrosis in patients with HFpEF and myocardial fibrosis. The secondary objectives are to determine the efficacy of pirfenidone compared to placebo with regard to improving ventricular structure and function, left atrial volume and function, aortic function, myocardial energetics, circulating markers of fluid status and myocardial injury, exercise tolerance and quality of life in patients with HFpEF and myocardial fibrosis. The study will also evaluate the safety of pirfenidone in patients with HFpEF and compare it to that of placebo, and record screening and recruitment data in order to inform a subsequent phase III study. Outcome measures are listed in full in Table 1.

**Table 1:** Primary and secondary outcome measures

Primary outcome	
Absolute change in myocardial ECM volume, measured using CMR, from baseline to week 52.	
Secondary outcome measures	
(a) Absolute change in LV and RV mass, volumes, ejection fraction and tissue characteristics from baseline to week 52, measured using CMR. (b) Absolute change in absolute myocardial ECM volume from baseline to week 52, measured using CMR.* (c) Absolute change myocardial cell volume from baseline to week 52, measured using CMR.* (d) Absolute change in LV diastolic function, strain, backscatter and torsion from baseline to week 52, measured using echocardiography. (e) Absolute change in LA and RA volume, and LA function from baseline to week 52, measured using CMR. (f) Absolute change in pulse wave velocity and aortic distensibility from baseline to week 52, measured using CMR. (g) Absolute change in myocardial energetic status (PCr/ATP ratio) from baseline to week 52, measured using ^31^P MRS. (h) Absolute change in NT-proBNP, and HS-troponin T from baseline to week 13, baseline to week 26 and baseline to week 52. (i) Absolute change in exercise tolerance from baseline to week 52, measured using 6-min walk distance. (j) Absolute change in health status (quality of life), HF symptoms and physical limitations from baseline to week 52, measured using change in KCCQ score. (k) All-cause mortality, cardiovascular mortality and hospitalisation for heart failure will be recorded but the trial is not powered for these clinical outcomes.	
Safety outcome measures	
(a) Treatment-emergent AEs, SAEs, SARs, SUSARs (b) Treatment-emergent changes in vital signs (c) Treatment-emergent changes in physical examination findings (d) Treatment-emergent changes in laboratory investigations (haematology and biochemistry) (e) Treatment-emergent changes in ECG	
Other outcome measures	
(a) Screening and recruitment data will be collected in order to inform the subsequent phase III study	

^*31*^*P MRS*^31^phosphorous magnetic resonance spectroscopy, *AE* adverse event, *ATP* adenosine triphosphate, *CMR* cardiac magnetic resonance, *ECG* electrocardiogram, *ECM* extracellular volume matrix, *HF* heart failure, *Hs-Troponin T* high-sensitivity troponin t, *KCCQ* Kansas City Cardiomyopathy Questionnaire, *LA* left atrial, *LV* left ventricular, *NT-proBNP* N-terminal pro brain natriuretic peptide, *PCr* phosphocreatine, *RA* right atrial, *RV* right ventricular, *SAE* serious adverse event, *SAR* serious adverse reaction, *SUSAR* serious unexpected serious adverse reaction

*See Online Appendix for calculations

### Patients

The eligibility criteria are summarised in Table 2. Briefly, patients are ≥ 40 years of age, have a LVEF ≥ 45%, have symptoms and signs of HF and have B-type natriuretic peptide (BNP) ≥ 100 pg/ml or N-terminal pro-B-type natriuretic peptide (NT-proBNP) ≥ 300 pg/ml at baseline (patients in atrial fibrillation at baseline are required to have BNP ≥ 300 pg/ml or NT-proBNP ≥ 900 pg/ml). In addition, in order to be randomised, patients are required to have myocardial fibrosis, defined as an ECM volume ≥ 27% measured using CMR at Visit 0. An ECM volume threshold of 27% was chosen because it represents one standard deviation above that in healthy volunteers scanned at the host institution (Manchester University NHS Foundation Trust). Patients who meet eligibility criteria but who have an ECM volume < 27% are invited to take part in a sub-study (see below) and are entered into a registry. Key exclusion criteria include a probable alternative cause of patients’ symptoms and contraindications to CMR scanning or gadolinium-based contrast agent administration, including severe renal dysfunction, defined as an estimated glomerular filtration rate of < 30 mL/min.

**Table 2:** Eligibility criteria

Inclusion criteria	
1. Written informed consent 2. Male or female, aged 40 years or older 3. HF, defined as one symptom present at the time of screening, and one sign present at the time of screening or in the previous 12 months. Symptoms and signs are defined as: Symptoms: dyspnoea on exertion, orthopnoea or paroxysmal nocturnal dyspnoea. Signs: peripheral oedema, crackles on chest auscultation post-cough, raised jugular venous pressure or chest x-ray demonstrating pleural effusion, pulmonary congestion, or cardiomegaly 4. LVEF ≥ 45% at visit 0, (any local LVEF measurement made using echocardiography or CMR). 5. BNP ≥ 100 pg/ml or NT-proBNP ≥ 300 pg/ml recorded at visit 0. For patients in atrial fibrillation on visit 0 ECG, BNP ≥ 300 pg/ml or NT-proBNP ≥ 900 pg/ml at visit 0. 6. In order to be randomised, patients must also have myocardial fibrosis, defined as ECM volume ≥ 27% by CMR at visit 0	
Exclusion criteria	
1. Myocardial infarction, coronary artery bypass graft surgery or percutaneous coronary intervention within the previous 6 months 2. Probable alternative cause of patient’s HF symptoms that in the opinion of the investigator primarily accounts for patient’s dyspnoea such as significant pulmonary disease, anaemia or obesity. Specifically, patients with the below are excluded: (a) Severe chronic obstructive pulmonary disease (COPD) (i.e. requiring home oxygen, chronic nebuliser therapy, or chronic oral steroid therapy), or (b) Haemoglobin < 9 g/dl, or (c) Body mass index (BMI) > 55 kg/m2 3. Known pericardial constriction, genetic hypertrophic cardiomyopathy, or infiltrative cardiomyopathy 4. Clinically significant congenital heart disease 5. Presence of severe valvular heart disease 6. Atrial fibrillation or flutter with a resting ventricular rate > 100 bpm 7. Any medical condition, which in the opinion of the Investigator, may place the patient at higher risk from his/her participation in the study, or is likely to prevent the patient from complying with the requirements of the study or completing the study 8. Severe renal dysfunction at visit 0, defined as eGFR < 30 mL/min (using CKD-EPI calculation), or end-stage renal disease requiring dialysis 9. History of severe hepatic impairment or liver dysfunction at visit 0, defined as total bilirubin above the ULN (excluding patients with Gilbert’s syndrome), AST or ALT > 3 times the ULN or alkaline phosphatase > 2.5 times the ULN 10. Prolonged corrected QT interval, defined as a corrected QT interval > 500 msec on ECG using Bazett formula 11. Known hypersensitivity to any of the components of the IMP 12. Use of other investigational drugs at the time of enrolment, or within 30 days or 5 half-lives of enrolment, whichever is longer 13. Fluvoxamine use within 28 days of visit 0 14. Contraindication to MRI scanning or gadolinium-based contrast agent 15. Pregnancy, lactation or planning pregnancy. Women of childbearing capacity are required to have a negative serum pregnancy test before treatment, must agree to pregnancy tests at study visits and home urine pregnancy tests, and must agree to maintain highly effective contraception during the study and for 3 months thereafter. Similarly, male participants with female partners of childbearing potential must agree to maintain highly effective contraception during the study and for 3 months thereafter.	

*ALT* alanine aminotransferase, *AST* aspartate transaminase, *BNP* brain natriuretic peptide, *CMR* cardiac magnetic resonance, *CKD-EPI* chronic kidney disease epidemiology collaboration, *ECG* electrocardiogram, *ECM* extracellular volume matrix, *eGFR* estimated glomerular filtration rate, *HF* heart failure, *IMP* investigational medicinal product, *LVEF* left ventricular ejection fraction, *MRI* magnetic resonance imaging, *NT-proBNP* N-terminal pro brain natriuretic peptide, *ULN* upper limit of normal

Recruitment to PIROUETTE began on March 7, 2017, after approval by a NHS Research Ethics Committee, the UK Medicines and Healthcare Products Regulatory Agency (MHRA) and the UK Health Research Authority (HRA). The study is being conducted in accordance with Good Clinical Practice and the Declaration of Helsinki.

### Study Design

**Fig. 1:**
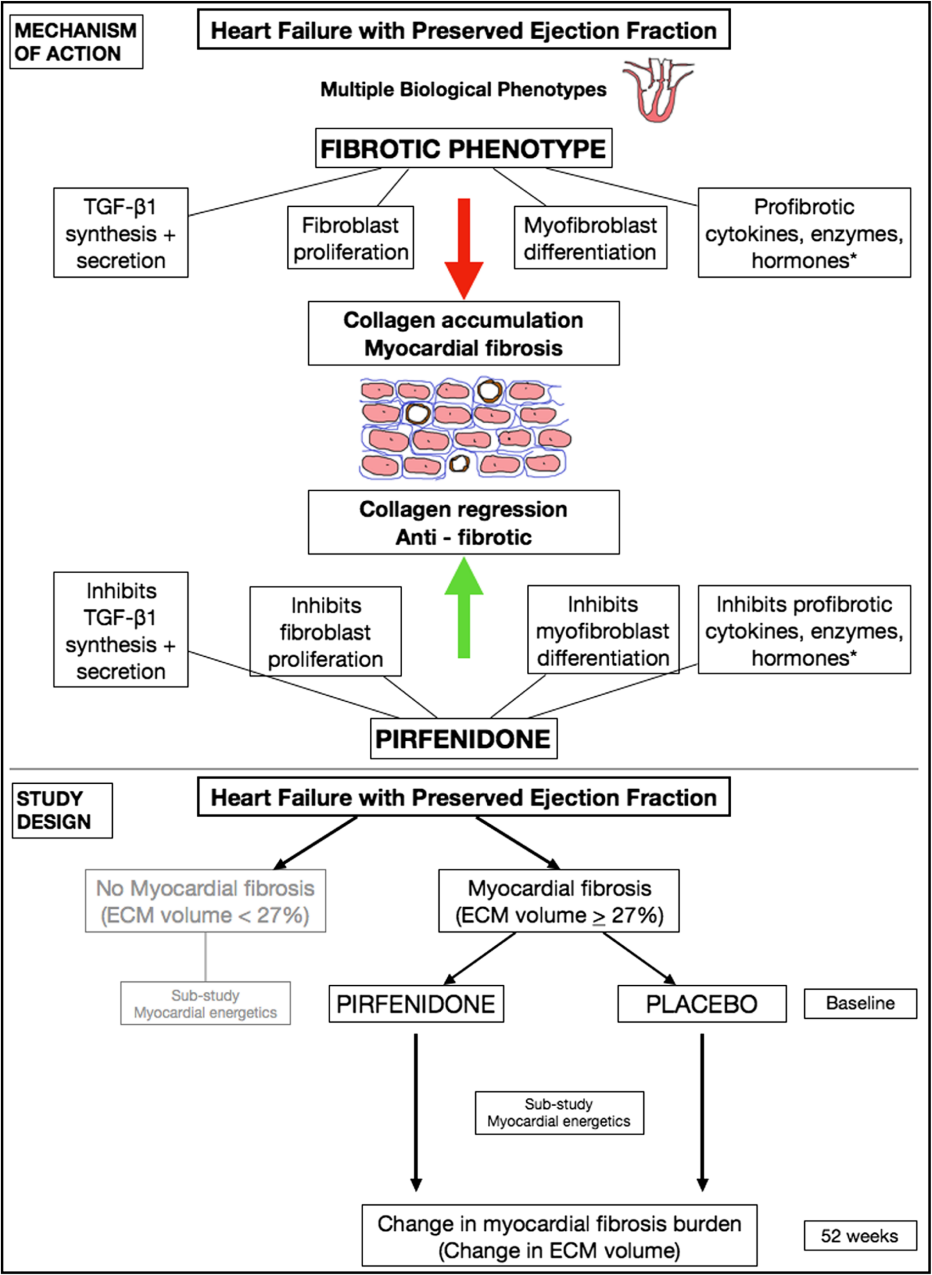
HFpEF pathophysiological mechanism being targets, mechanism of action of pirfenidone and study schematic. Asterisk indicates MMPs, TIMPs, interleukins, renin-angiotensin-aldosterone system

The study design is summarised in Fig. 1.

#### Baseline Evaluations

Potential participants are identified at four NHS hospital trusts in the North West of England, UK (see Online Appendix), and are invited to a baseline visit. At the baseline visit, participants are consented and undergo assessment of eligibility criteria, review of medical history and medications, assessment of vital signs, physical examination, biochemistry and haematological laboratory investigations, electrocardiogram (ECG), CMR, echocardiogram, 6-min walk test and the Kansas City Cardiomyopathy Questionnaire (KCCQ). Details of these procedures are provided in the Online Appendix.

#### Randomisation

After confirmation of eligibility, participants are randomised in a 1:1 ratio to double-blind treatment with either pirfenidone or placebo. Randomisation is done using web randomisation software accessed using a secure website provided via the Clinical Trials Unit. Block randomisation, stratified by sex (because ECM volume is higher in females than males), is implemented, with computer generated randomisation allocations.

#### Investigational Medicinal Product

The active treatment is pirfenidone (Esbriet) 2403 mg daily, taken orally as three 267 mg capsules three times per day. The comparator is placebo (manufactured to appear identical to pirfenidone 267 mg capsules), taken as three capsules three times per day. After treatment with IMP is started, it is titrated, as tolerated, to the full dose of three capsules three times a day over a 14-day period, as follows: Days 1 to 7: one capsule, three times a day; days 8 to 14: two capsules, three times a day; day 15 onward: three capsules, three times a day. In participants who experience side effects, the IMP dose may be reduced, and subsequently re-escalated as appropriate. Every effort is made to maintain patients on the optimal dose (i.e. 9 capsules per day). The treatment period is 52 weeks.

A target dose of pirfenidone of 2403 mg daily was chosen because it proved clinically effective and safe in pulmonary fibrosis [35–37]. A treatment duration of 52 weeks was chosen because it is in keeping with the trials in pulmonary fibrosis, and, based on previous work with renin-angiotensin-aldosterone system inhibitors, it was felt to represent the minimum period within which meaningful fibrosis regression can occur [24–27].

#### Safety Monitoring and Follow-up

Follow-up visits are conducted at week 1 (telephone interview) and weeks 2, 4, 8, 13, 17, 21, 26, 39 and 52 (all in-person). Unscheduled visits can occur at the investigators discretion (for example, an adverse event making it necessary to assess the participant in clinic). At follow-up visits, patients undergo a review of symptoms and concomitant medications, assessment of vital signs, physical examination, biochemistry and haematological laboratory investigations and an ECG. At the final visit (week 52), baseline procedures are repeated in order to assess the primary and secondary outcome measures. Participants who request to withdraw from the trial early undergo ‘exit’ data collection, equivalent to the final visit, provided they have received at least 6 months of IMP, for use in a sensitivity analysis. With specific consent, an additional blood sample is taken at baseline, 13, 26 and 52 weeks and stored in a central biorepository for future analysis.

### Sub-Study

Whilst myocardial fibrosis is an important pathophysiological mechanism in HFpEF, the mechanisms by which it exerts a deleterious effect are not clear. A widely held hypothesis is that myocardial fibrosis impairs myocyte capillary blood supply and causes arteriolar vasomotor dysfunction, which leads to energy starvation of cells and impaired energetics. Indeed, there is evidence of impaired myocardial energetics in HFpEF, but the relationship between ECM expansion and myocardial energy metabolism has not been investigated [52]. The hypotheses for the sub-study are (1) at baseline, myocardial ECM volume will be inversely associated with phosphocreatine (PCr) to adenosine triphosphate (ATP) ratio; and (2) pirfenidone-induced ECM regression will be associated with an improvement in PCr:ATP ratio.

PCr:ATP ratio will be measured at baseline using ^31^phosphorus magnetic resonance spectroscopy (^31^P MRS) in a subgroup of patients who are due to be randomised (i.e. all have an ECM volume ≥ 27%) and a subgroup of patients without ECM expansion (i.e. ECM volume < 27%) but who otherwise meet eligibility criteria, in order to assess the relationship between myocardial ECM volume and energetic status. ^31^P MRS will then be repeated in randomised patients after 52 weeks of treatment in order to compare the change in energetic status in the pirfenidone group with the change in energetic status in the placebo group. The relationships between myocardial ECM volume, energetic status and mechanical properties will be investigated. Details of the ^31^P MRS procedure are given in the Online Appendix.

### Protocol Amendments

Modifications to the PIROUETTE protocol are summarised in Table 1 in the Online Appendix.

### Study Management and Committees

PIROUETTE is conducted by the research team, under the guidance of C.A.M. (Chief Investigator), in conjunction with Liverpool Clinical Trials Research Centre (CTRC), which is a UK Clinical Research Collaboration fully registered Clinical Trials Unit. The sponsor is Manchester University NHS Foundation Trust. The trial is funded by the UK National Institute for Health Research (NIHR). Roche Products Limited has gifted the IMP. NIHR and Roche Products Limited have had no role in the study design other than through their external peer review processes. A trial steering committee (TSC) provides overall supervision for the trial and provides advice through its independent Chairman. An independent data and safety monitoring committee (IDSMC) is responsible for reviewing and assessing recruitment, interim monitoring of safety and effectiveness, trial conduct and external data and submits periodic reports to the TSC. Further details are in the Online Appendix.

## Statistical Considerations

### Sample Size

Thirty-seven participants per group are required to detect an absolute minimum difference, between pirfenidone and placebo groups, of 2% in terms of change in CMR ECM volume from baseline following 52 weeks of treatment, with 80% power at a 5% significance level (2-sided), assuming a standard deviation of the within-patient differences from baseline equal to 3%, as per Garg et al. [53]. This effect size is based on a conservative estimate of the magnitude of ECM regression that is expected to translate into improved clinical outcomes, based on the magnitude of histological collagen regression (3.6% absolute reduction) seen with 52 weeks of treatment with losartan, a medication known to improve clinical outcomes in patients with HF with reduced ejection fraction, in patients with hypertensive heart disease and baseline ECM expansion [25]. To allow for treatment discontinuation in up to 20% of participants prior to final follow-up, the number randomised to each group will be inflated to 47. This discontinuation rate is in keeping with the proportion of patients who discontinued pirfenidone prematurely, at the same target dose as here, in the trials in pulmonary fibrosis [35–37]. Therefore, 94 participants are required to undergo randomisation. In the previously described study by Schelbert el al, 63% of patients with HFpEF had an ECM volume ≥ 27% [22]. Thus, and in order to allow for variation in the proportion of participants with an ECM volume ≥ 27%, it is anticipated that up to 200 patients may need to be recruited to undergo baseline assessment.

### Sub-Study Sample Size

Thirty-three participants per group are required to detect an absolute minimum difference in PCr/ATP ratio of 0.37 between ECM expansion and no ECM expansion groups at baseline (80% power, 5% significance level, 2-sided), assuming a standard deviation of the between group differences of 0.52, as per Phan [52]. This effect size is based on that seen in previous studies [52, 54]. Twenty-six participants per group are required to detect an absolute minimum difference, between pirfenidone and placebo groups, of 0.4 in terms of absolute change in PCr/ATP ratio from baseline following 52 weeks of treatment (80% power, 5% significance level, 2-sided), assuming a standard deviation of the within-patient differences from baseline equal to 0.5, as per Beadle [55]. This effect size is based on that seen in other studies [54–57]. In order to allow for a potential 20% drop out prior to final scan at 52 weeks, the number scanned at baseline will be inflated to 33 per group.

### Analysis

The trial will be analysed and reported using the ‘Consolidated Standard of Reporting Trials’ (CONSORT) and the International Conference on Harmonisation E9 guidelines. All primary analyses will be on an intention to treat basis including all randomised participants retained in their randomised treatment groups. Secondary causal analyses (according to dose and duration of intervention) will also be undertaken to assess the causal impact of treatment received. Analyses of covariance will be used to compare myocardial ECM volume (and other measures) between pirfenidone and placebo groups, adjusting for baseline ECM volume. The conventional 5% significance level will be used.

For the sub-study, PCr/ATP ratio and mechanical variables will be compared between patients with and without ECM expansion at baseline using an independent *t* test assuming the measurements are normally distributed, with transformation as necessary. Correlation analysis will be used to assess the relationships between PCr/ATP ratio, mechanics and ECM volume. PCr/ATP ratio will be compared between pirfenidone and placebo groups using analyses of covariance, adjusting for baseline PCr/ATP ratio.

## Discussion

‘The path forward to improve HF trials needs the connecting of biological pathways, drug mechanisms of action, and underlying pathophysiology’ [1].

HFpEF is a diverse syndrome that involves multiple pathophysiological mechanisms [58]. Indeed, the biologically heterogeneity is cited as a reason for the failure of the clinical effectiveness trials to date in HFpEF, and the need for interventions that target specific underlying biological mechanisms has become well recognised [1–3, 59]. The phase II PIROUETTE trial has been designed in order to target ‘the right patient population’, with ‘the right intervention’, using ‘the right clinical end points’, in order to maximise the chances of successfully modulating an important disease mechanism, and thus, if the results are positive, maximise the chances of translating this into phase III success [2].

The PIROUETTE trial specifically targets HFpEF patients with myocardial fibrosis. Myocardial fibrosis is a key pathophysiological mechanism of HFpEF and myocardial fibrotic burden is strongly and independently associated with adverse outcome in HFpEF [22]. Recruitment to PIROUETTE is determined by individual patient myocardial fibrotic burden.

The intervention, pirfenidone, is an anti-fibrotic agent that leads to substantial regression of myocardial fibrosis in preclinical models, and has proven clinical effectiveness in pulmonary fibrosis. Importantly from a mechanistic point of view for this study and for the wider cardiovascular field, pirfenidone does not have a haemodynamic effect. Myocardial fibrosis regression has been observed in humans following interventions with haemodynamic effects, both drug and mechanical, but, to our knowledge, myocardial fibrosis regression has not been observed in humans with a ‘dedicated’ anti-fibrotic, agent i.e. without haemodynamic effect [24–27, 60]. Thus, the results of the PIROUETTE trial will provide fundamental insight into cardiovascular pathophysiology.

Choice of primary outcome variable in phase II HF trials has proven challenging [1]. For example, while natriuretic peptides are of established prognostic value, they are not reflective of specific pathophysiological mechanisms, and therefore do not provide feedback on whether or not an intervention has modulated the mechanism it was designed to target. At least in part as a result, interventions associated with improvements in natriuretic peptide levels at phase II have often not translated into improved clinical outcomes at phase III. Moreover, recent trials have failed to show improvement in clinical outcomes with natriuretic peptide-guided care [61]. The primary outcome variable in PIROUETTE, change in myocardial ECM volume, is of proven prognostic value in HFpEF and, importantly, is both specific to the myocardial pathophysiological mechanism that the intervention is designed to target, and sensitive to biological response [31, 60].

Elevated circulating natriuretic peptide levels are required for study entry in order to increase the diagnostic confidence of HF [62]. A LVEF ≥ 45% is used because it is in keeping with other contemporary HFpEF trials [11, 63]. Other structural measures, such as left ventricular hypertrophy or left atrial dilatation, are not required for entry because of their variable association with HFpEF, and their inconsistent association with outcome in HFpEF [58]. Baseline CMR scanning will serve to exclude specific causes of HF in the context of a normal or near-normal EF, such as cardiac amyloidosis, which potentially account for a quarter of patients with a label of ‘HFpEF’, and which may have confounded previous HFpEF trials [28, 64].

The sub-study is designed to provide additional mechanistic insight into the pathophysiology of HFpEF, the action of pirfenidone and, more broadly, the myocardial fibrosis paradigm. The data generated by the sub-study will provide additional support for a subsequent phase III study, if the primary outcome is reached.

## Conclusions

The PIROUETTE trial will evaluate the efficacy and safety of pirfenidone in patients with HFpEF and myocardial fibrosis. By targeting a known prognostically important mechanism of HFpEF, i.e. myocardial fibrosis, with an intervention designed to modulate this mechanism, using a primary outcome measure specific to this mechanism and sensitive to its biological modulation, and patient recruitment personalised to individual expression of this mechanism, PIROUETTE has been designed to overcome the disconnect between phase II and III HF trials and maximise the chances of both successfully modulating an important mechanism of HFpEF, and translating the findings, if positive, into phase III success. As such, PIROUETTE could serve as a blueprint for future phase II HF trials.

## Electronic Supplementary Material

ESM 1 (DOCX 53 kb)
