# Supplementary material for: Pirfenidone in Heart Failure with Preserved Ejection Fraction—Rationale and Design of the PIROUETTE Trial
Source: Cardiovasc Drugs Ther. 2019 May 8;33(4):461–70. doi: 10.1007/s10557-019-06876-y (PMC6689029; doi:10.1007/s10557-019-06876-y)
Supplement: Supplementary file 1 — (DOCX 53 kb) [file 10557_2019_6876_MOESM1_ESM.docx]

**Data Supplement**

**Trial oversight committees**

*Steering Committee:* Andrew L Clark (Chair), Roy Gardner, Catriona Graham

*Independent Data and Safety Monitoring Committee:* Richard Emsley (Chair), Scot Garg, Mamas Mamas.

**Clinical trials unit**

The trial is being conducted in conjunction with Liverpool Clinical Trials Research Centre (CTRC). The CTRC is part of the Liverpool Clinical Trials Collaborative, which is a UK Clinical Research Collaboration (UKCRC; [www.ukcrc.org](http://www.ukcrc.org)) fully registered Clinical Trials Unit (CTU). The CTU are responsible for trial management and monitoring, including trial conduct, regulatory approvals, data management, randomisation and statistical analysis.

**Participating sites**

Wythenshawe Hospital, Manchester University NHS Foundation Trust

Manchester Royal Infirmary, Manchester University NHS Foundation Trust

Salford Royal Hospital, Salford Royal NHS Foundation Trust

Macclesfield District General Hospital, East Cheshire NHS Foundation Trust

Stepping Hill Hospital, Stockport NHS Foundation Trust

**Procedures**

Cardiac Magnetic Resonance (CMR) Imaging

CMR is performed on a 1.5T scanner (Siemens Healthcare, Germany). CMR imaging includes the following:

*Cine imaging*

Standard cine imaging with steady-state free precession (SSFP) includes 2-chamber, 3-chamber, 4-chamber, ventricular short-axis and atrial short-axis acquisitions. Left and right ventricular volumes, ejection fraction, left ventricular (LV) mass, wall thickness, ventricular long-axis function and atrial volumes are measured using dedicated software (CVI_42_, Circle Cardiovascular Imaging Inc., Canada).

*Late Gadolinium enhancement*

Late gadolinium enhancement (LGE) imaging is performed 10 minutes after a 0.15 mmol/kg intravenous bolus of gadoterate meglumine (Dotarem, Guerbet) with a phase sensitive inversion recovery sequence matching the cine imaging planes.

Ischaemic and non-ischaemic LGE is quantified from the ventricular short-axis stack using a full-width half-maximum threshold and is expressed in grams and as a percentage of the left ventricle.(1)

*Extracellular matrix (ECM) volume*

T1 mapping (by MOdified Look-Locker Inversion recovery [MOLLI]) is performed before and 15 minutes after gadolinium contrast agent administration on basal and mid ventricular short-axis slices. T1 maps are manually contoured as described previously. (2, 3) Infarcted myocardium on LGE imaging is excluded. ECM volume is calculated as: ECM volume = (1 – haematocrit) [ΔR1_myocardium_] / [ΔR1_bloodpool_] (26), where ΔR1 is the difference in relaxation rates (1 / T1) pre-contrast and post-contrast.(4) Haematocrit is measured on the day of scanning.

Absolute myocardial ECM volume and absolute myocardial cell volume are calculated as follows.(5)

Total myocardial volume (ml) = left ventricular mass (g) / specific gravity of myocardium (1.05 g/ml)

Absolute myocardial ECM volume (ml) = Total myocardial volume (ml) x ECM volume

Absolute myocardial cell volume (ml) = Total myocardial volume (ml) – absolute ECM volume (ml)

*Aortic function*

SSFP cine images are acquired as an aortic ‘candy-stick’ view and at the level of the pulmonary artery bifurcation perpendicular to the ascending and descending aorta. Through-plane phase contrast velocity-encoded acquisitions are performed at the same ascending and descending aortic positions.

Pulse-wave velocity (PWV) is calculated using the transit-time method as follows.(6)

*PWV = δx/δt,*

Where δx is the distance between the ascending and descending aorta slice positions and δt is the transit time delay of the systolic flow curve in the descending aorta relative to the ascending aorta. The transit time (δt) is measured as the time difference from the midpoints (half the time to peak flow) of the systolic upslope flow curves.(7)

Aortic distensibility is measured using the method described by Vogues et al.(7) The maximum (A_max_) and minimum (A_min_) cross-sectional area of the ascending aorta is measured from a 40-phase cine acquisition at the level of the pulmonary artery. Blood pressure is taken at the time of image acquisition (P_systolic_ = systolic blood pressure, P_diastolic_ = diastolic blood pressure). Aortic distensibility is calculated as follows:

*Aortic distensibility (10^-3^ mmHg^-1^) = (A_max_-A_min_) / A_min_ x (P_systolic_ - P_diastolic_)*

*Other outcome measures*

T2 mapping, using a T2-prepared-SSFP sequence, is performed before contrast administration on basal and mid ventricular short-axis slices, then averaged.

^31^Phosphorous Cardiac Magnetic Resonance Spectroscopy (^31^P-MRS)

^31^P-MRS is performed on a 3.0T scanner (Siemens Healthcare, Germany) equipped with a ^31^P/^1^H surface coil (Rapid Biomedical GMBH, Rimpar, Germany). After localization, real-time pilot cines are acquired in the four-chamber and short axis planes.^31^P-MRS sequences are then acquired with a 3D chemical shift imaging (CSI) sequence. The CSI grid is positioned over the real-time cine images to cover the septal myocardium in the four-chamber and short-axis views.

Spectra are analysed on jMRUI (Java magnetic resonance user interface, developed by A. Van den Boogaart, Katholieke Universiteit Leuven, Leuven, Belgium).(8, 9) Acquired spectra are summed, allowing for post-processing with 15Hz Lorezian line broadening and phase correction. The phosphocreatinine (PCr) peak is set as the zero-reference point. Quantification is performed using AMARES (advanced method for accurate, robust and efficient spectral fitting). Phosphocreatine (PCr) to adenosine triphosphate (ATP) ratio is calculated as the ratio of area under the curve (AUC). This value is corrected for blood contamination by subtracting one sixth of the 2,3-diphosphoglycerate (2,3-dpg) AUC from the ATP AUC.(10) Values are corrected for the effects of partial saturation by applying a correction factor calculated using the flip angle for a TR time of 1.5s, assuming T1 values of PCr and gamma-ATP as 3.8 seconds and 2.4 seconds respectively.(11)

Echocardiography

Echocardiography is performed on a Vivid e95 system (General Electric (GE) Healthcare, USA) and post-processing performed on Echopacs software (GE Vingmed Ultrasound, Norway).

Mitral valve flow (E and A wave velocity, E/A ratio, deceleration time) and tissue Doppler annular velocities (septal and medial) are measured from apical 4-chamber view as per British Society of Echocardiography guidelines.(12) Measurements are made in at least 3 cardiac cycles and averaged.

LV strain and strain rate is measured using speckle-tracking analysis in the apical 4-chamber, apical 3-chamber, and apical 2-chamber views. Torsion is calculated using speckle-tracking analysis in the basal- and apical-short-axis. Left atrial (LA) strain is measured using speckle-tracking analysis in the apical 4-chamber and apical 2-chamber views. Three consecutive cardiac cycles are recorded and averaged as image quality allows. The onset of the QRS complex is used as the ventricular reference point.(13) Speckle-tracking is not performed if image quality is inadequate, defined as >1 segment image dropout of the LV or LA.

The components of LA strain are defined as:

LA reservoir strain = peak LA strain

LA booster strain = LA strain at the onset of the P-wave

LA conduit strain = LA reservoir strain – LA booster strain.

Patients in atrial fibrillation do not have LA booster strain measurements, therefore LA reservoir strain equals LA conduit strain.(14)

Calibrated integrated backscatter (cIB) is calculated by measuring the tissue intensity of the anteroseptum, inferolateral wall, and pericardium in the parasternal long-axis view. Mean cIB is calculated by subtracting mean pericardial integrated backscatter intensity at end-diastole from mean integrated backscatter intensity from the anteroseptum and inferolateral wall, which are then averaged to calculate mean cIB.(15)

6-Minute Walk Test

The 6-minute walk test is performed as per recommendations by the American Thoracic Society.(16) Overall distance is recorded to the nearest metre.

Kansas City Cardiomyopathy Questionnaire (KCCQ)

KCCQ is completed as standard and the results are calculated by the CTRC.(17)

Blood testing

Biochemistry and haematology blood tests for safety monitoring are processed in the laboratory at the Wythenshawe Hospital site. NT-pro BNP and hs-Troponin T are analysed centrally on a Cobas e411 analyser (Roche Diagnostic, West Sussex, UK).

Bio-repository

With specific patient consent, additional blood samples are stored in a central biorepository for planned future ethically approved research. This will include analysis of growth differentiating factor-15 (GDF-15).

**Supplemental Table 1. Major protocol modifications implemented following protocol amendment and rationale for change**

| Amendment number | Release date | Modifications | Change rationale |
| --- | --- | --- | --- |
| 1 | 13/3/18 | Modified inclusion and exclusion criteria: |  |
|  |  | The time frame in which elevated natriuretic peptides are required was extended from Visit 0 to Visit 0 or within the previous 12 months, as follows:  BNP ≥ 100pg/ml or NTproBNP ≥ 300pg/ml at Visit 0 or within past 12 months. For patients in atrial fibrillation at Visit 0 ECG, BNP ≥ 300pg/ml or NTproBNP ≥ 900pg/ml at Visit 0 or within past 12 months. | To ensure that patients with HFpEF in whom natriuretic peptide levels have fallen with treatment (e.g. diuretics) are not excluded. |
|  |  | Lower natriuretic peptide thresholds were included for patients who have been admitted to hospital with heart failure in the previous 12 months, as follows:  Heart failure admission within the past 12 months and BNP ≥ 60pg/ml or NTproBNP ≥ 200pg/ml at Visit 0 or within past 12 months. For patients in atrial fibrillation, BNP ≥ 200pg/ml or NTproBNP ≥ 600pg/ml at Visit 0 or within past 12 months | To ensure that patients with HFpEF in whom natriuretic peptide levels have fallen with treatment (e.g. diuretics) are not excluded, and to bring this criterion more in line with other on going trials.(18) |
|  |  | The exclusion criterion regarding liver impairment was split into two separate exclusion criteria, as follows:  History of severe hepatic impairment.  Liver dysfunction at Visit 0, defined as total bilirubin above the ULN (excluding patients with Gilbert’s syndrome), AST or ALT >3 times the ULN or alkaline phosphatase >2.5 times the ULN. | To improve clarity |

**References**

1. Flett AS, Hasleton J, Cook C, Hausenloy D, Quarta G, Ariti C, et al. Evaluation of techniques for the quantification of myocardial scar of differing etiology using cardiac magnetic resonance. JACC Cardiovascular imaging. 2011;4(2):150-6.

2. Wong TC, Piehler K, Meier CG, Testa SM, Klock AM, Aneizi AA, et al. Association between extracellular matrix expansion quantified by cardiovascular magnetic resonance and short-term mortality. Circulation. 2012;126(10):1206-16.

3. Wong TC, Piehler KM, Kang IA, Kadakkal A, Kellman P, Schwartzman DS, et al. Myocardial extracellular volume fraction quantified by cardiovascular magnetic resonance is increased in diabetes and associated with mortality and incident heart failure admission. European heart journal. 2014;35(10):657-64.

4. Messroghli DR, Moon JC, Ferreira VM, Grosse-Wortmann L, He T, Kellman P, et al. Clinical recommendations for cardiovascular magnetic resonance mapping of T1, T2, T2* and extracellular volume: A consensus statement by the Society for Cardiovascular Magnetic Resonance (SCMR) endorsed by the European Association for Cardiovascular Imaging (EACVI). 2017:1-24.

5. Treibel TA, Kozor R, Schofield R, Benedetti G, Fontana M, Bhuva AN, et al. Reverse Myocardial Remodeling Following Valve Replacement in Patients With Aortic Stenosis. Journal of the American College of Cardiology. 2018;71(8):860-71.

6. Ibrahim el SH, Johnson KR, Miller AB, Shaffer JM, White RD. Measuring aortic pulse wave velocity using high-field cardiovascular magnetic resonance: comparison of techniques. J Cardiovasc Magn Reson. 2010;12:26.

7. Voges I, Jerosch-Herold M, Hedderich J, Pardun E, Hart C, Gabbert DD, et al. Normal values of aortic dimensions, distensibility, and pulse wave velocity in children and young adults: a cross-sectional study. J Cardiovasc Magn Reson. 2012;14:77.

8. Naressi A, Couturier C, Castang I, de Beer R, Graveron-Demilly D. Java-based graphical user interface for MRUI, a software package for quantitation of in vivo/medical magnetic resonance spectroscopy signals. Comput Biol Med. 2001;31(4):269-86.

9. Naressi A, Couturier C, Devos JM, Janssen M, Mangeat C, de Beer R, et al. Java-based graphical user interface for the MRUI quantitation package. Magma. 2001;12(2-3):141-52.

10. Conway MA, Bottomley PA, Ouwerkerk R, Radda GK, Rajagopalan B. Mitral regurgitation: impaired systolic function, eccentric hypertrophy, and increased severity are linked to lower phosphocreatine/ATP ratios in humans. Circulation. 1998;97(17):1716-23.

11. Tyler DJ, Emmanuel Y, Cochlin LE, Hudsmith LE, Holloway CJ, Neubauer S, et al. Reproducibility of 31P cardiac magnetic resonance spectroscopy at 3 T. NMR in biomedicine. 2009;22(4):405-13.

12. Wharton G, Steeds R, Allen J, Phillips H, Jones R, Kanagala P, et al. A minimum dataset for a standard adult transthoracic echocardiogram: a guideline protocol from the British Society of Echocardiography. Echo Res Pract. 2015;2(1):G9-G24.

13. Mor-Avi V, Lang RM, Badano LP, Belohlavek M, Cardim NM, Derumeaux G, et al. Current and evolving echocardiographic techniques for the quantitative evaluation of cardiac mechanics: ASE/EAE consensus statement on methodology and indications endorsed by the Japanese Society of Echocardiography. Eur J Echocardiogr. 2011;12(3):167-205.

14. Freed BH, Daruwalla V, Cheng JY, Aguilar FG, Beussink L, Choi A, et al. Prognostic Utility and Clinical Significance of Cardiac Mechanics in Heart Failure With Preserved Ejection Fraction: Importance of Left Atrial Strain. Circ Cardiovasc Imaging. 2016;9(3).

15. Jellis C, Wright J, Kennedy D, Sacre J, Jenkins C, Haluska B, et al. Association of imaging markers of myocardial fibrosis with metabolic and functional disturbances in early diabetic cardiomyopathy. Circ Cardiovasc Imaging. 2011;4(6):693-702.

16. Laboratories ATSCoPSfCPF. ATS statement: guidelines for the six-minute walk test. Am J Respir Crit Care Med. 2002;166(1):111-7.

17. Green CP, Porter CB, Bresnahan DR, Spertus JA. Development and evaluation of the Kansas City Cardiomyopathy Questionnaire: a new health status measure for heart failure. Journal of the American College of Cardiology. 2000;35(5):1245-55.

18. Solomon SD, Rizkala AR, Gong J, Wang W, Anand IS, Ge J, et al. Angiotensin Receptor Neprilysin Inhibition in Heart Failure With Preserved Ejection Fraction: Rationale and Design of the PARAGON-HF Trial. JACC Heart failure. 2017;5(7):471-82.
